# Huperzine A Alleviates Mechanical Allodynia but Not Spontaneous Pain via Muscarinic Acetylcholine Receptors in Mice

**DOI:** 10.1155/2015/453170

**Published:** 2015-12-01

**Authors:** Zhen-Xing Zuo, Yong-Jie Wang, Li Liu, Yiner Wang, Shu-Hao Mei, Zhi-Hui Feng, Maode Wang, Xiang-Yao Li

**Affiliations:** ^1^Department of Neurosurgery, First Affiliated Hospital, Medical College, Xi'an Jiaotong University, Xi'an, Shaanxi 710061, China; ^2^Center for Mitochondrial Biology and Medicine, The Key Laboratory of Biomedical Information Engineering of Ministry of Education, School of Life Science and Technology and Frontier Institute of Science and Technology, Xi'an Jiaotong University, Xi'an 710049, China; ^3^Institute of Neuroscience and Collaborative Innovation Center for Brain Science, School of Medicine, Zhejiang University, Hangzhou, Zhejiang 310058, China

## Abstract

Chronic pain is a major health issue and most patients suffer from spontaneous pain. Previous studies suggest that Huperzine A (Hup A), an alkaloid isolated from the Chinese herb *Huperzia serrata*, is a potent analgesic with few side effects. However, whether it alleviates spontaneous pain is unclear. We evaluated the effects of Hup A on spontaneous pain in mice using the conditioned place preference (CPP) behavioral assay and found that application of Hup A attenuated the mechanical allodynia induced by peripheral nerve injury or inflammation. This effect was blocked by atropine. However, clonidine but not Hup A induced preference for the drug-paired chamber in CPP. The same effects occurred when Hup A was infused into the anterior cingulate cortex. Furthermore, ambenonium chloride, a competitive inhibitor of acetylcholinesterase, also increased the paw-withdrawal threshold but failed to induce place preference in CPP. Therefore, our data suggest that acetylcholinesterase in both the peripheral and central nervous systems is involved in the regulation of mechanical allodynia but not the spontaneous pain.

## 1. Introduction

Chronic pain affects 15–18% of the population [1]. As well as allodynia, hyperalgesia, and spontaneous pain, patients with chronic pain also present with cognitive impairment, emotional change, insomnia, and mood disorders [2]. Currently, analgesic drugs are used primarily to treat pain [3] but limited effects are a major issue for clinical management [4]. Therefore, finding new drugs is important for the treatment of chronic pain.

Herbal medicines are a potential source of analgesic drugs. The use of herbal medicines has long history, and the analgesic effects of several of them have been evaluated by using extracts or isolated compounds [5]. Among these, Huperzine A (Hup A), an alkaloid isolated from a Chinese club-moss, has received much attention due to its potent and selective inhibition of acetylcholinesterase [6–10], and its analgesic effects have been evaluated in both normal animals and models of spinal cord injury (Table 1) [11–13]. Spontaneous pain, which occurs without stimulation, has been reported to affect ~96% of chronic pain patients [14], and this is the primary target of clinical pain management [15, 16]. However, whether Hup A affects spontaneous pain is not clear. Therefore, we designed the current study to evaluate the analgesic effects of Hup A on spontaneous pain using the conditioned place preference (CPP) behavioral assay [17].

## 2. Materials and Methods

### 2.1. Animals

Male C57B L/6 mice aged 8–10 weeks (20–35 g) were housed four or five per cage at constant room temperature (25 ± 1°C) and relative humidity (60 ± 5%) under a 12 h light/dark schedule (lights on 07.00–19.00), with food and water available* ad libitum*. Before the behavioral tests, the mice were allowed to adapt to laboratory conditions for about one week and to habituate to the testing situation for at least 15 min before experiments. To induce inflammatory pain, 10 *μ*L of 50% complete Freund's adjuvant (CFA; Sigma, St. Louis, MO) was injected subcutaneously into the plantar surface of the left hindpaw. The Animal Care and Use Committee of Zhejiang University approved all of the mouse protocols.

### 2.2. Common Peroneal Nerve (CPN) Model

The CPN ligation model of neuropathic pain was generated as described previously [18, 19]. Briefly, mice were anesthetized with isoflurane (1–3%, as needed). The left CPN between the anterior and posterior muscle groups was slowly ligated with chromic gut suture 5-0 (Ethicon, Blue Ash, USA) until the appearance of twitching of the digits. The skin was sutured using 5-0 silk and cleaned with povidone iodine. Sham surgery was conducted in the same manner but the nerve was not ligated. Animals were kept in a normal cage after surgery. The mice were used for behavioral tests on postsurgical days 3–14.

### 2.3. Mechanical Allodynia Test

On the experimental day, the von Frey behavioral test was performed according to the up–down algorithm described by Dixon [20]. To determine reflex responses evoked by mechanical stimuli, animals were placed on a raised mesh grid and covered with a clear plastic box for containment. Calibrated von Frey filaments were applied to the middle of the plantar surface of each paw until the filament bent. Brisk withdrawal or paw flinching was considered a positive response. Lifting of the paw due to normal locomotor behavior was ignored. In the absence of a response, the filament of next greater force was applied. Following a response, the filament of the next lower force was applied. The tactile stimulus producing a 50% likelihood of a withdrawal response was calculated and treated as the paw-withdrawal threshold (PWT). The PWTs of mice were normalized by the PWTs tested before the sham or nerve-injury operations.

### 2.4. Cannulation and Microinjection

The cannula surgery and microinjection were performed as described previously [19]. Briefly, mice were anesthetized with isoflurane (1–3%, as needed) in 100% oxygen at 0.5  L/min via face-mask. The scalp was shaved and cleaned with iodine (Triadine, Shanghai, China) and alcohol. The head was fixed into an adapter mounted on a stereotaxic frame (model 962; Kopf, California, USA) and AKWA Tears (Akorn, Buffalo Grove, IL, USA) were applied to the eyes. An incision was made over the skull and the surface was exposed. Two small holes were drilled above the anterior cingulate cortex (ACC), and the dura was gently reflected. Guide cannulas were placed 0.7 mm anterior to bregma, 0.3 mm lateral to the midline, and 0.75 mm ventral to the surface of the skull. For microinjection, each mouse was restrained in a plastic cone (Braintree Scientific, Braintree, USA), and a small hole was cut in the plastic overlying the microinjection guides. Each dummy cannula was removed, and a microinjection cannula was inserted into each guide. A 30-gauge injection cannula was inserted to a depth 0.7 mm deeper than each guide. Huperzine A (0.5 *μ*L, 0.01 *μ*g/*μ*L) was delivered at 0.5 *μ*L/min using a syringe driven by an infusion pump (Harvard Apparatus, Inc., South Natick, MA). The volume delivered was confirmed by watching the movement of the meniscus in a length of calibrated polyethylene tubing (PE10, Braintree Scientific, Braintree, USA). After delivery to one side of the brain, the cannula was left in place for 1 min to prevent solution from flowing back up the guide. The cannula was then retracted and inserted into the opposite side of the brain. Ten minutes after microinjection, the mice were given the mechanical allodynia test.

### 2.5. Conditioned Place Preference (CPP) Test

The CPP test was adapted from the paradigm established by King et al. in adult rats [17, 21]. Briefly, mice were preconditioned for three days, starting 3 days after CPN ligation, and the chamber preference was evaluated on preconditioned day 3. A single trail conditioning was performed as below: the following day (day 7 after CPN), mice received the appropriate control (i.e., vehicle) paired with a randomly chosen chamber in the morning, and the appropriate drug paired with the other chamber 4 h later (in the afternoon). Chamber pairings were counterbalanced. Twenty hours after the afternoon pairing, mice were placed in the CPP box with access to all chambers and their behavior recorded for 15 min was analyzed for chamber preference. The preference time was calculated as the time spent in the drug-paired chamber minus the time spent in the saline-paired chamber.

The multitrial conditioning was performed as follows: preconditioning to an automated 3-chamber CPP box was performed across 3 days, starting 1 day after CFA injection. All animals are exposed to the environment with full access to all chambers across 30 min each day. On day 3, behavior was recorded for 15 min and analyzed to verify absence of preconditioning chamber preference. Animals spending more than 80% (time spent > 720 sec) or less than 20% (time spent < 120) of the total time in a chamber were eliminated from further testing. Following the preconditioning phase, mice underwent conditioning across 6 days with alternating treatment-chamber pairings. Mice received vehicle- (e.g., saline-) chamber pairing on odd days and Hup A-chamber pairing on even days. Mice were placed in the paired chamber with no access to the other chamber immediately following vehicle or drug. Drug and chamber pairing were counterbalanced. On test day animals were placed into the neutral chamber and had access to all chambers during the 15 min observation period, during which time spent in each of the chambers was recorded.

### 2.6. Novel Object Recognition Test

The novel object recognition test was adapted from the paradigm reported by Leger et al. [22]. Briefly, mice were put into a plastic box (40 cm × 40 cm × 40 cm) to habituate for 5 min. Twenty-four hours later, two identical bottles were placed 10 cm from two corners of the box, and mice were allowed to explore them for 5 min. Twenty-four hours later, one of the bottles was replaced by a new bottle with a different shape and mice were again allowed to explore freely for 5 min, and the times spent exploring the old and new bottles were recorded. The discrimination index was calculated as the difference between the times spent with the new and old bottles.

### 2.7. Acetylcholinesterase (AChE) Activity

The AChE activity was determined using an assay kit and following the manufacturer's recommendations (MAK119; Sigma, St. Louis, USA). Briefly, 0.1 mg/kg Hup A was injected intraperitoneally into mice after 3 days of CPN ligation, and the ACCs were sampled after 0, 0.5, 2, and 6 h. The AChE activity was normalized to the 0 h injection group.

### 2.8. Data Analysis

SigmaPlot 11.0 was used to plot and fit the data. Statistical comparisons were made using Student's* t*-test, the paired* t*-test, and one-way or two-way repeat measure ANOVA (Two-way RM ANOVA); the Student-Newmann-Keuls (SNK) or Tukey's test was used for* post hoc* comparisons. All data are presented as the mean SEM. In all cases, *P* < 0.05 was considered statistically significant.

## 3. Results

### 3.1. Analgesic Effects of Hup A on Mechanical Allodynia under Chronic Pain Conditions

**Figure 1:**
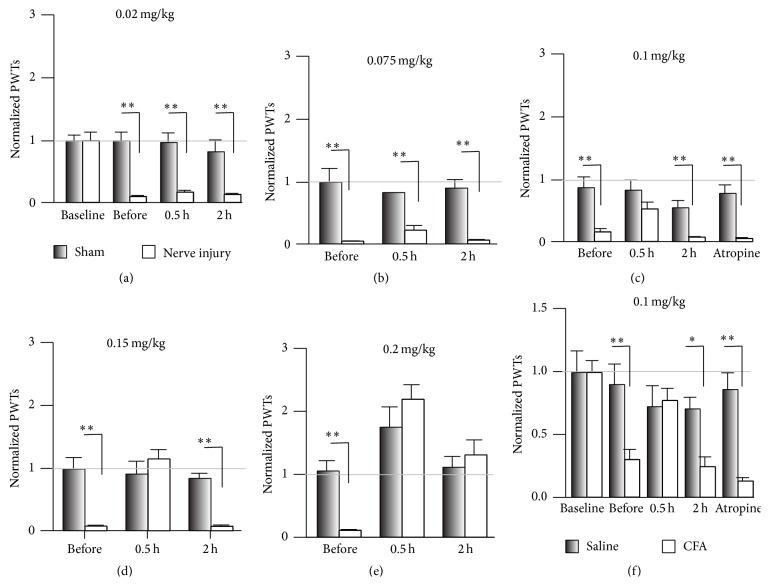
Systemic injection of Hup A raised the PWT in nerve-injured mice. (a) Hup A at 0.02 mg/kg had no effect on the PWTs in the sham and nerve-injury groups (two-way repeated measures ANOVA, sham versus injury: *F*_1;55_ = 50.04, *P* < 0.01; treatments: *F*_3;55_ = 8.17, *P* < 0.01, *n* = 7 per group, ^*∗∗*^ *P* < 0.01 under Tukey's test). (b) Hup A at 0.075 mg/kg had no effect on the PWTs in the sham and nerve-injury groups (two-way repeated measures ANOVA, sham versus injury: *F*_1;32_ = 227.45, *P* < 0.01; treatments: *F*_2;32_ = 0.08, *P* > 0.01, *n* = 5 for sham, *n* = 6 for CPN, ^*∗∗*^ *P* < 0.01 under Tukey's test). (c) Hup A at 0.1 mg/kg increased the PWTs in the nerve-injury group, but not in the sham group, and this effect was blocked by atropine (two-way RM ANOVA, sham versus injury: *F*_1;69_ = 14.60, *P* < 0.01; treatments: *F*_4;69_ = 20.13, *P* < 0.01, *n* = 7 per group, ^*∗∗*^ *P* < 0.01 under Tukey's test). (d) Hup A at 0.15 mg/kg raised the PWTs in the nerve-injury group, but not in the sham group (two-way RM ANOVA, sham versus injury: *F*_1;29_ = 29.91, *P* < 0.01; treatments: *F*_2;29_ = 9.18, *P* < 0.01, *n* = 5 per group, ^*∗∗*^ *P* < 0.01 under Tukey's test). (e) Increasing the dose of Hup A to 0.2 mg/kg raised the PWTs in both groups, and the analgesic effects lasted >2 h (two-way RM ANOVA, sham versus injury: *F*_1;41_ = 0.29, *P* > 0.05; treatments: *F*_2;41_ = 23.17, *P* < 0.01, *n* = 7 per group, ^*∗∗*^ *P* < 0.01 under SNK test). (f) Hup A at 0.1 mg/kg increased the PWTs in the CFA injection group, but not in the saline group, and this effect was blocked by atropine (two-way RM ANOVA, saline versus CFA: *F*_1;94_ = 13.24, *P* < 0.01; treatments: *F*_4;94_ = 6.89, *P* < 0.01, saline, *n* = 10; CFA, *n* = 9, ^*∗*^ *P* < 0.05; ^*∗∗*^ *P* < 0.01 under Tukey's test). “Baseline” indicates the PWTs before operation. “Before” indicates PWTs before intraperitoneal drug injection.

Yu et al. reported that Hup A attenuates the mechanical allodynia induced by static intrathecal compression [12], suggesting that Hup A is a good candidate pain-killer. Here we used the CPN ligation model, which causes little impairment of motor function [18], to further evaluate the analgesic effects of Hup A on neuropathic pain. The PWT was tested before and three days after CPN ligation, and the ligation significantly decreased it (sham versus nerve injury, *n* = 7 per group, Tukey's test, *P* < 0.001; Figure 1(a)). A low dose of Hup A (0.02 mg/kg and 0.075 mg/kg, i.p.; Figures 1(a) and 1(b)) did not change the PWT, while a higher dose (0.1 mg/kg and 0.15 mg/kg) increased the PWTs of mice with nerve injury to normal levels at 0.5 h after injection. The hypersensitivity returned 2 h after injection (Tukey's test, *P* < 0.001; Figures 1(c) and 1(d)). While Hup A at 0.2 mg/Kg increased the PWTs of mice from both the sham and nerve-injury groups, the analgesic effect lasted for >2 h (sham versus nerve injury, Tukey's test, *P* > 0.05; Figure 1(e)). To investigate whether muscarinic acetylcholine receptors (mAChRs) are involved in the analgesic effects of Hup A, atropine (1 mg/kg), an antagonist of mAChRs, was injected first, and Hup A (0.1 mg/kg) was injected 0.5 h later. Under these conditions, atropine blocked the effects of Hup A on the PWTs (sham versus nerve injury, Tukey's test, *P* < 0.001; Figure 1(c)), suggesting that mAChRs are involved in the regulation of mechanical allodynia. Similar to previous reports [12], our data suggest that Hup A alleviates mechanical allodynia.

To investigate whether Hup A has an analgesic effect on chronic inflammatory pain, we injected CFA into the left hindpaw, and this decreased the PWTs one day after injection (Baseline: saline versus CFA, Tukey's test, *P* > 0.05; after injection: Tukey's test, *P* < 0.01; Figure 1(f)). Injection of Hup A (0.1 mg/kg, i.p.) increased the PWTs to the control level (saline versusCFA, Tukey's test, *P* > 0.05), and this effect did not last for 2 h (saline versus CFA, Tukey's test, *P* < 0.05). Similarly, injection of atropine (1 mg/kg, i.p.) blocked the effect of Hup A on the PWTs (atropine + Hup A, saline versus CFA, Tukey's test, *P* < 0.001). Therefore, our data suggested that Hup A alleviates the mechanical allodynia of neuropathic and chronic inflammatory pain via mAChRs.

### 3.2. Effects of Hup A on Spontaneous Pain

**Figure 2:**
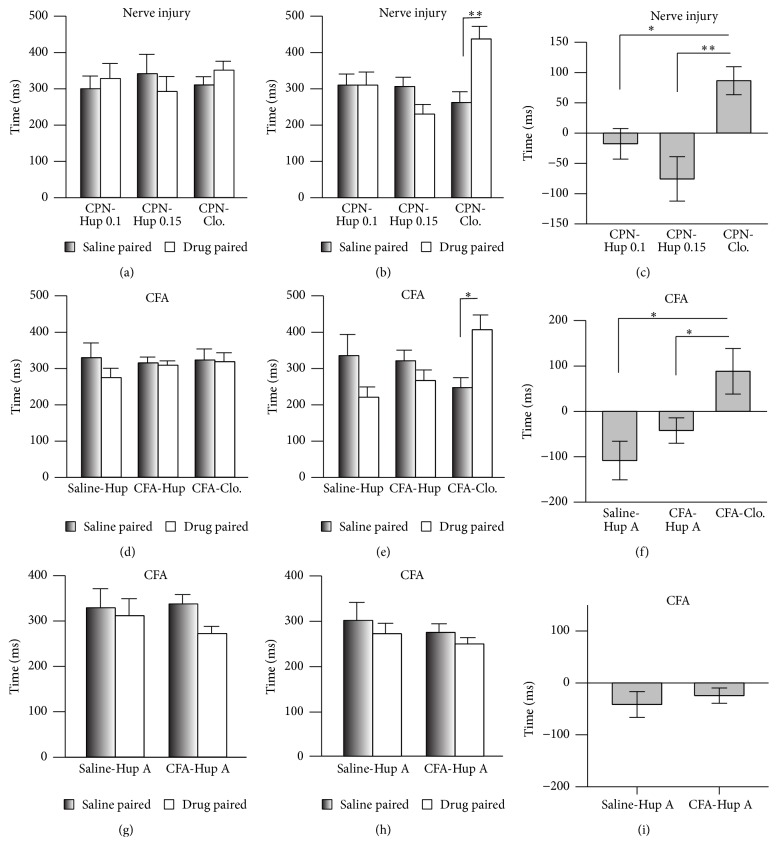
Systemic injection of clonidine but not Hup A alleviated spontaneous neuropathic pain as assessed in the CPP test. (a) Times spent in the chambers during the preconditioning period (two-way RM ANOVA, groups: *F*_2;33_ = 1.30, *P* > 0.05; saline versus drug: *F*_1;33_ = 0.03, *P* > 0.05, *n* = 6 for Hup 0.1, clonidine group, *n* = 5 for Hup 0.15). (b) Clonidine but not Hup A (0.1 mg/kg and 0.15 mg/kg) induced preference for the drug-paired chamber (two-way RM ANOVA, groups: *F*_2;33_ = 11.79, *P* < 0.01; saline versus drug: *F*_1;33_ = 0.98, *P* > 0.05, *n* = 6 for Hup 0.1, clonidine group, *n* = 5 for Hup 0.15, ^*∗*^ *P* < 0.01 under SNK test). (c) Preference times induced by Hup A and clonidine in mice with nerve injury (one-way ANOVA, *F*_2;16_ = 8.50, *P* < 0.01, *n* = 6 for Hup 0.1, clonidine group, *n* = 5 for Hup 0.15, ^*∗*^ *P* < 0.05 under SNK test). (d) Time spent by mice in the chambers in the saline- and CFA-injected groups during the preconditioning period (two-way RM ANOVA, groups: *F*_2;57_ = 0.54, *P* > 0.05; saline versus drug: *F*_1;57_ = 0.82,*P* > 0.05, *n* = 5 for saline-Hup A, *n* = 16 for CFA-Hup A, *n* = 9 for CFA-Clo.). (e) Clonidine but not Hup A induced preference for the drug-paired chamber by CFA-injected mice (two-way RM ANOVA, groups: *F*_2;57_ = 3.34, *P* = 0.05; saline versus drug: *F*_1;57_ = 0.01, *P* > 0.05, *n* = 5 for saline-Hup A, *n* = 16 for CFA-Hup A, and *n* = 9 for CFA-Clo.; ^*∗*^ *P* < 0.05 under SNK test). (f) Preference times of mice with CFA injection induced by Hup A and clonidine (one-way ANOVA, *F*_2;28_ = 5.13, *P* < 0.05, ^*∗*^ *P* < 0.05 under SNK test). (g) Time spent by mice in the chambers in the saline- and CFA-injected groups during the preconditioning period for multitrial conditioning (two-way RM ANOVA, groups: *F*_1;29_ = 0.86, *P* > 0.05; saline versus drug: *F*_1;29_ = 1.15, *P* > 0.05, *n* = 7 for saline-Hup A, *n* = 8 for CFA-Hup A). (h) Hup A (0.1 mg/kg) did not induce preference for the drug-paired chamber by CFA-injected mice in the multitrial conditioning (two-way RM ANOVA, groups: *F*_1;29_ = 1.43, *P* > 0.05; saline versus drug: *F*_1;29_ = 0.93, *P* > 0.05, *n* = 7 for saline-Hup A, *n* = 8 for CFA-Hup A). (i) Preference times of mice with CFA injection induced by Hup A in the multitrial conditioning (*t*-test, *P* > 0.05).

Spontaneous pain is one of the major pathological phenomena of chronic pain [15, 16]. Here, we used the CPP assay [17] to evaluate the effects of Hup A on spontaneous pain. The mice did not show place preference in the preconditioning test (Figure 2(a)), and the injection of clonidine (0.5 mg/Kg, i.p.) into the nerve-injured mice induced a preference for the drug-paired chamber (*n* = 6, *P* < 0.05; Figure 2(b)), suggesting the presence of spontaneous pain induced by CPN ligation. But Hup A (0.1 mg/Kg and 0.15 mg/kg) did not induce place preference in nerve-injured mice (Figure 2(b)). However, the effects of clonidine were markedly different from those of Hup A (groups: *F*_2;33_ = 11.79, *P* < 0.01, two-way repeated measures ANOVA, *n* = 6 for Hup A 0.1 mg/kg and clonidine group, *n* = 5 for Hup A 0.15 mg/kg; Figure 2(b)). The preference time for clonidine also differed from that of Hup A (one-way ANOVA, *F*_2;16_ = 8.50, *P* < 0.01, *n* = 6 for Hup 0.1, clonidine group, *n* = 5 for Hup 0.15, Figure 2(c)). These data suggested that Hup A at the dosage of 0.1 mg/kg and 0.15 mg/kg does not alleviate spontaneous pain in mice with CPN ligation.

Peripheral inflammation may induce spontaneous pain, so we used the same behavioral paradigm to test the effects of Hup A on ongoing pain. Similarly, clonidine (i.p.) increased the time spent in the drug-paired chamber (saline-paired versus clonidine-paired, *n* = 9, *P* < 0.05; Figures 2(d) and 2(e)), while Hup A (0.1 mg/Kg) did not show any effect (Figures 2(d) and 2(e)). The preference time for clonidine also differed from that of Hup A (*F*_2;28_ = 5.13, *P* < 0.05, one-way ANOVA; Figure 2(f)). To further confirm these results, a multitrial conditioning was employed, in which mice received Hup A for several times, and the multiple application of Hup A still did not induce place preference (Figures 2(g)–2(i)). Therefore our data suggested that Hup A has no effect on the spontaneous pain induced by peripheral inflammation.

### 3.3. Ambenonium Chloride Has No Effect on Spontaneous Pain

**Figure 3:**
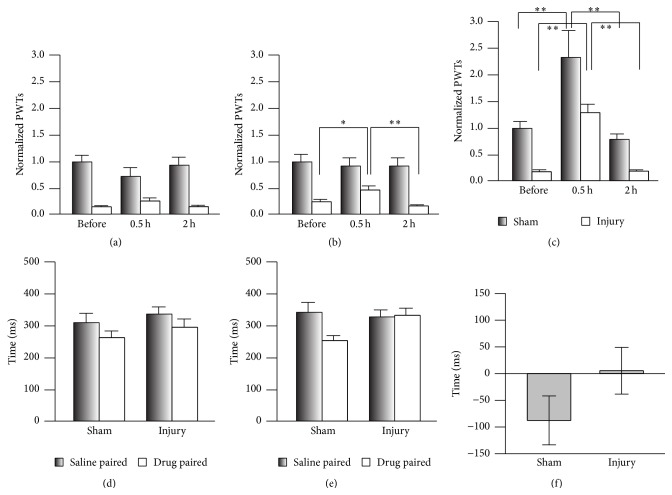
Application of ambenonium chloride had no effect on spontaneous pain. (a) Ambenonium at 0.01 mg/kg had no effect on the PWTs of the sham and nerve-injury groups (two-way RM ANOVA, sham versus injury: *F*_1;35_ = 30.45, *P* < 0.01; treatments: *F*_2;35_ = 0.59, *P* > 0.05, *n* = 6 per group). (b) Ambenonium at 0.05 mg/kg increased the PWTs of the nerve-injury group (two-way RM ANOVA, sham versus injury: *F*_1;41_ = 27.52, *P* < 0.01; treatments: *F*_2;41_ = 2.94, *P* > 0.05, *n* = 6 for sham, *n* = 8 for injury; ^*∗*^ *P* < 0.05 and ^*∗∗*^ *P* < 0.01 under SNK test). (c) Increasing the dose of ambenonium to 0.1 mg/kg raised the PWTs in both groups (two-way RM ANOVA, sham versus injury: *F*_1;32_ = 28.57, *P* < 0.01; treatments: *F*_2;32_ = 22.42, *P* < 0.01, ^*∗∗*^ *P* < 0.01 under Tukey's test). (d) Time spent in the chambers during the CPP preconditioning period (two-way RM ANOVA, sham versus injury: *F*_1;43_ = 4.21, *P* > 0.05; saline versus drug: *F*_1;43_ = 1.76, *P* > 0.05, *n* = 9 for sham group, *n* = 13 for injury group). (e) Ambenonium at 0.05 mg/kg did not induce preference for the drug-paired chamber in the CPP test (two-way RM ANOVA, sham versus injury: *F*_1;43_ = 11.57, *P* < 0.05; saline versus drug: *F*_1;43_ = 1.61, *P* > 0.05, *n* = 9 for sham group, *n* = 13 for injury group). (f) No difference in the preference time induced by ambenonium at 0.05 mg/kg was detected in the CPP test (*t*-test, *P* > 0.05, *n* = 9 for sham group, *n* = 13 for injury group).

Since both peripheral and central sensitization are involved in the regulation of chronic pain [23], Hup A applied systemically may alleviate the mechanical allodynia by inhibiting AChE [24] in the peripheral nerve system. To test this, we investigated the analgesic effects of ambenonium chloride, a competitive AChE inhibitor that does not pass through the blood-brain barrier (BBB) [25, 26]. Ambenonium at 0.01 mg/kg (i.p.) had no effect on the PWTs (Figure 3(a)), while it increased them in the nerve-injury group at 0.05 mg/kg (Figure 3(b)) and in both groups at 0.1 mg/kg (Figure 3(c)). We therefore used ambenonium at 0.05 mg/kg to investigate the involvement of AChE in the regulation of spontaneous pain. The mice did not show a preference for the ambenonium-paired chamber in CPP (Figures 3(d)–3(f)), and no difference was detected in the preference time between the sham and nerve-injury groups (Figure 3(f)), suggesting that ambenonium has no effect on spontaneous pain. Therefore, inhibiting AChE in the peripheral nervous system alleviates evoked pain but not spontaneous pain.

### 3.4. Analgesic Effects of Hup A in the Anterior Cingulate Cortex

**Figure 4:**
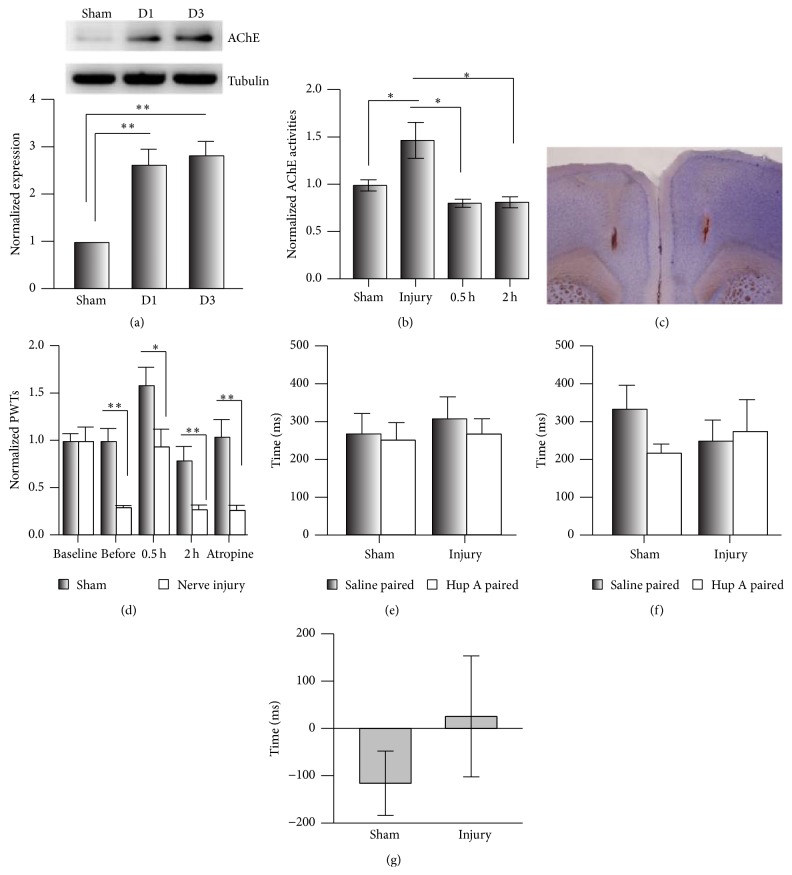
Infusion of Hup A into the anterior cingulate cortex did not alleviate spontaneous neuropathic pain. (a) The expression levels of AChE in the ACC were increased by the nerve injury (one-way ANOVA, *F*_2;14_ = 14.64, *P* < 0.01; *n* = 5 per group, ^*∗∗*^ *P* < 0.05 under SNK test). (b) The AChE activity in the ACC of mice with nerve injury was increased, and this was inhibited by Hup A at 0.1 mg/kg, i.p. (one-way ANOVA, *F*_3;26_ = 5.81, *P* < 0.01; *n* = 10 for sham group, *n* = 9 for injury, *n* = 4 for 0.5 h group and 2 h group, ^*∗*^ *P* < 0.05 under SNK test). (c) An example showing the injection site in the ACC of hematoxylin and eosin stained brain section. (d) Infusion of Hup A into the ACC increased PWTs in the sham and nerve-injury groups, and atropine blocked this analgesic effect (two-way RM ANOVA, sham versus injury: *F*_1;49_ = 14.89, *P* < 0.01; treatments: *F*_4;49_ = 15.93, *P* < 0.01, *n* = 5 per group, ^*∗∗*^ *P* < 0.01 under Tukey's test). (e) Time spent in the chambers during the preconditioning period of the CPP test (two-way RM ANOVA, sham versus injury: *F*_1;15_ = 0.39, *P* > 0.05; saline versus drug: *F*_1;15_ = 0.27, *P* > 0.05, *n* = 4 per group). (f) Infusion of Hup A into the ACC did not induce a preference for the drug-paired chamber in the CPP test (two-way RM ANOVA, sham versus injury: *F*_1;15_ = 0.09, *P* > 0.05; saline versus drug: *F*_1;15_ = 0.40, *P* > 0.05, *n* = 4 per group). (g) No change occurred in the preference time induced by Hup A in the CPP (*t*-test, *P* > 0.05).

Although it has been reported that Hup A passes through the BBB, whether the AChE in the central nervous system is decreased by Hup A was unclear. The ACC is important in the maintenance of chronic pain [19, 21]. We first evaluated the expression levels of AChE on day 1 (D1) and day 3 (D3) after CPN ligation and found that in the nerve-injury group it increased to 2.63 ± 0.34 and 2.83 ± 0.31 times that of the sham group, respectively (*n* = 5 per group, Figure 4(a)). Similarly, the activities of AChEs at D3 increased to 1.47 ± 0.19 times that of the Sham group, and Hup A (0.1 mg/kg) decreased them to the level of the sham group at 0.5 h after injection (0.81 ± 0.04, *n* = 4) and 2 h (0.82 ± 0.06, *n* = 4, one-way ANOVA, *F*_3;26_ = 5.81, *P* < 0.01, Figure 4(b)). These data suggested that the systemic injection of Hup A decreases the activities of AChE in the ACC.

We further infused Hup A into the ACC (0.005 *μ*g/0.5 *μ*L/side) (Figure 4(c)). This markedly increased the PWTs in both sham and nerve-injured mice (sham: before versus Hup A, *P* < 0.01; nerve injury: before versus Hup A, *P* < 0.01, *n* = 5 for both groups), while this effect was blocked by atropine (i.p., sham versus nerve injury, *P* < 0.001; Figure 4(d)), suggesting the involvement of cingulate mAChRs in pain regulation. While infusion of Hup A into the ACC did not induce place preference, mice in both of the sham (paired *t*-test, *P* > 0.05, *n* = 4/group) and nerve-injury groups (paired* t*-test, *P* > 0.05, *n* = 4/group) spent equal times in the chambers during preconditioning (Figure 4(e)). Furthermore, when Hup A was infused, the mice did not show a preference for the drug-paired chamber (two-way RM ANOVA, Figure 4(f)). Also, no difference was detected between the sham and nerve-injury groups in the preference time (*t*-test, *P* > 0.05; Figure 4(g)). Our data suggested that AChE in the ACC is involved in the regulation of mechanical allodynia, but not spontaneous pain.

**Figure 5:**
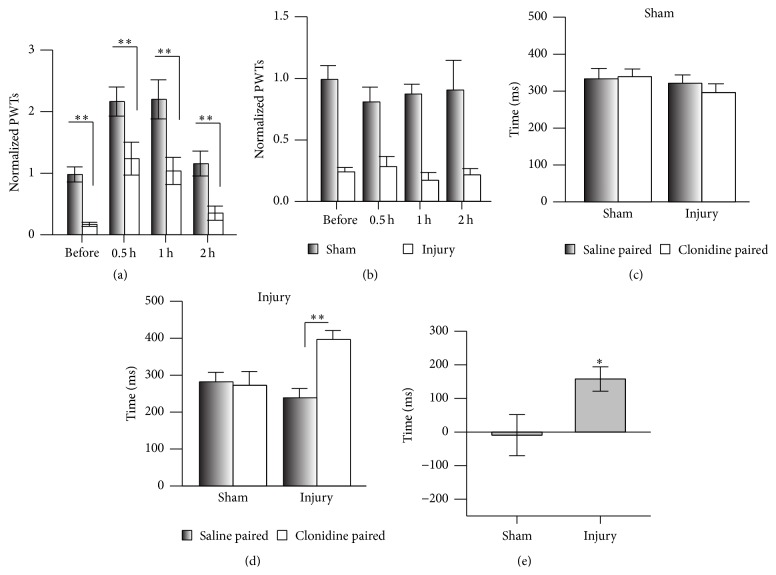
Infusion of clonidine into the anterior cingulate cortex alleviated spontaneous neuropathic pain. (a) Infusion of clonidine into the ACC increased the PWTs in the sham and nerve-injury groups (two-way RM ANOVA, sham versus injury: *F*_1;63_ = 21.12, *P* < 0.01; treatments: *F*_3;63_ = 23.13, *P* < 0.01, *n* = 7 for sham, *n* = 9 for injury, ^*∗∗*^ *P* < 0.01 under Tukey's test). (b) Infusion of saline into the ACC had no effect on the PWTs (two-way RM ANOVA, sham versus injury: *F*_1;39_ = 44.54, *P* < 0.01; treatments: *F*_3;39_ = 0.29, *P* > 0.01, *n* = 5 per group). (c) Time spent in the chambers during the preconditioning period in the CPP test (two-way RM ANOVA, sham versus injury: *F*_1;31_ = 3.54, *P* > 0.05; saline versus drug: *F*_1;31_ = 0.10, *P* > 0.05, *n* = 8 per group). (d) Infusion of clonidine into the ACC induced a preference for the drug-paired chamber in the CPP test (two-way RM ANOVA, sham versus injury: *F*_1;31_ = 4.13, *P* = 0.06; saline versus drug: *F*_1;31_ = 4.06, *P* > 0.05, *n* = 8 per group, ^*∗∗*^ *P* < 0.01 under Tukey's test). (e) Hup A changed the preference time in the CPP test (*t*-test, *n* = 8 per group ^*∗*^ *P* < 0.05).

To exclude the possibility that the infusion damaged the ACC, which could lead to the negative performance of mice in the CPP, we infused clonidine (4 *μ*g/0.5 *μ*L/side) into the ACC and evaluated its analgesic effects on both PWT and CPP. Clonidine increased the PWTs in both sham and nerve-injured mice (two-way RM ANOVA, Figure 5(a)), while saline had no effect (Figure 5(b)). Similarly, the clonidine did not induce a clear preference on the sham mice (two-way RM ANOVA, Figure 5(c)), while a clear preference for the clonidine-paired chamber was evident on the injury mice (two-way RM ANOVA, Figure 5(d)), and the sham and nerve-injury groups were similar in the preference times due to the big variation of sham group (sham versus injury,* t*-test, *P* > 0.05, Figure 5(e)). Therefore, clonidine infused into the ACC attenuates both mechanical allodynia and spontaneous pain.

### 3.5. Effects of Clonidine and Hup A on Learning

**Figure 6:**
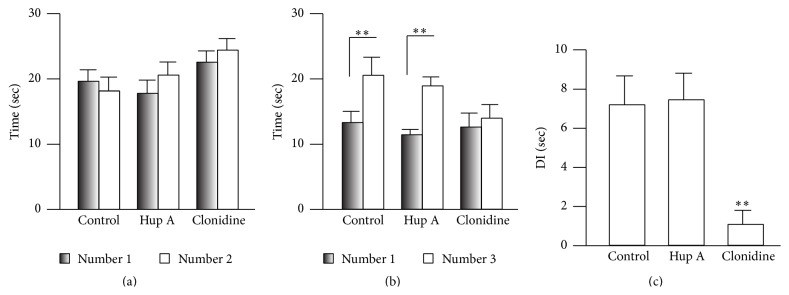
Hup A did not affect learning in mice. (a) Exploration times for the two identical bottles in each group (two-way RM ANOVA, objects: *F*_1;43_ = 2.19, *P* > 0.05; groups: *F*_1;43_ = 1.86, *P* > 0.05, *n* = 7 for control and clonidine group, *n* = 8 for Hup A group). (b) The control and Hup A groups spent more time exploring the novel object (two-way RM ANOVA, objects: *F*_1;43_ = 1.04, *P* > 0.05; groups: *F*_1;43_ = 51.05, *P* < 0.01, *n* = 7 for control and clonidine group, *n* = 8 for Hup A group, ^*∗∗*^ *P* < 0.01 under SNK test). (c) The discrimination index did not differ between the control and Hup A groups (one-way ANOVA, *F*_2;21_ = 7.10, *P* < 0.01; ^*∗*^ *P* < 0.05 under SNK test). DI indicates discrimination index.

Learning is involved in the performance of CPP, so the failure of Hup A to affect CPP may have been due to impaired learning. We therefore examined this possibility using the novel object recognition task. After 5 min habituation, two identical bottles (1 and 2) were put into symmetrical locations in the box, and the mice were allowed to explore them freely for 5 min, when they spent almost the same time exploring each bottle (two-way RM ANOVA, *F*_2;43_ = 1.86, *P* > 0.05, *n* = 7 for both control and clonidine groups, *n* = 8 for the Hup A group; Figure 6(a)). To avoid motor impairment during the training phase, the Hup A or clonidine was injected (i.p.) immediately after training. One bottle (#2) was replaced by a new one (#3) 24 h after training, and the times spent exploring the familiar (#1) and novel bottles (#3) were recorded. Surprisingly, the mice injected with Hup A (0.1 mg/kg) showed a clear preference for the novel bottle (#1: 11.61 ± 0.82 sec; #3: 19.12 ± 1.36 sec; Figure 6(b)), similar to the control group (#1: 13.49 ± 1.70 sec; #3: 20.74 ± 2.77 sec), but the clonidine group did not show a preference (#1: 12.82 ± 2.12 sec; #3: 14.15 ± 2.10 sec). And a significant difference was detected between the control (7.25 ± 1.48, *n* = 7) and clonidine groups (0.06 ± 0.03, *n* = 7, *P* < 0.05 versus control), but not the Hup A group (7.51 ± 1.35, *n* = 8, *P* > 0.05 versus control) on the discrimination index (one-way ANOVA, *F*_2;21_ = 7.10; *P* < 0.01, Figure 6(c)). These data suggested that the application of Hup A has no effect on learning in mice.

## 4. Discussion

In the current study, we evaluated the analgesic effects of Hup A on both mechanical allodynia and spontaneous pain in mice. Our data showed that although mechanical allodynia was significantly attenuated, spontaneous pain did not change when Hup A was injected systemically or infused locally into the ACC. Furthermore, we found that Hup A did not impair learning in the novel object recognition task. Ambenonium chloride, an inhibitor of AChE, had effects similar to Hup A. Our data therefore suggest that mAChRs are involved in the regulation of stimulation-evoked pain but not spontaneous pain.

### 4.1. Analgesic Effects of Hup A on Evoked Pain

The data from our study agrees with the previous report [12] that Hup A attenuates mechanical allodynia, a form of evoked pain induced by nerve injury. However, unlike that report, in which only rats with nerve injury were used [12], here we evaluated the analgesic effects of Hup A on mice with sham treatment or CPN ligation, and our data suggested that Hup A at 0.1 mg/kg (i.p.) is the appropriate dose for chronic pain management, because higher doses such as 0.2 mg/kg raised the PWTs of the mice in the sham group. The antinociceptive action of Hup A has also been evaluated using the hot-plate test in normal mice, which showed that Hup A at 70 *μ*g/kg increases the response latency [11]. This dose of Hup A is lower than that used in the current study and may be due to the different strain [27]. Furthermore, we showed for the first time that Hup A increased the PWTs of mice with peripheral inflammation. Therefore, our data suggested that Hup A alleviates the mechanical allodynia induced by both peripheral inflammation and nerve injury.

### 4.2. Effects of Hup A on Spontaneous Pain

Hup A evidently had no effect on spontaneous pain. The presence of spontaneous pain has been reported and evaluated using the CPP behavioral assay [17]. In our study, mice with nerve injury or peripheral inflammation did not show a preference for the Hup A-paired chamber, suggesting that spontaneous pain does not change. This was not due to the experimental design, because clonidine in the ACC did induce a preference for the drug-paired chamber in the nerve-injured mice [17]. Also, Hup A did not affect performance in the novel object-recognition behavioral paradigm, suggesting that the learning in mice is normal when Hup A is administered. It has been proposed that the reward system is involved in CPP [28]. We did find that mechanical allodynia was attenuated by Hup A, and the activity of AChE in the ACC was inhibited by Hup A (i.p.), which excluded the possibility that Hup A had no effect on the central nervous system. It is possible that the aversive state induced by nerve injury was not changed by Hup A, so mice did not show a preference for the drug-paired chamber. Whether the mAChR system is involved in regulating the reward system needs further study.

It has been shown that intrathecal Hup A increases the thermal escape latency and decreases flinching behavior in rats in the formalin test, which suggests that Hup A affects thermal allodynia and spontaneous pain [11]. However, it must be noted that flinching in the formalin test in that study was observed for several hours after injection, while, in our system, the spontaneous pain was evaluated several days after nerve injury or CFA injection, so the mechanisms mediating the spontaneous pain in the two models may be quite different.

### 4.3. AChE in Both the Peripheral and Central Nervous Systems Is Involved in the Regulation of Chronic Pain

Our data showed that the analgesic effect of Hup A was blocked by atropine, suggesting that the activity of mAChRs has a role in the regulation of evoked pain (Figure 1(b)). Hup A at 0.1 mg/kg only increased the PWTs of mice with nerve injury, but not the sham group, suggesting that mice with nerve injury are more sensitive to Hup A than the sham group, and this may be due to the increased AChEs under chronic pain conditions (Figure 4(a)). Since ambenonium chloride cannot pass through the BBB [25, 26], its analgesic effects suggest that peripheral AChE is involved in the hypersensitivity, while not ruling out its involvement in the central nervous system, since we also found that the activity of AChE in the ACC was inhibited by intraperitoneal Hup A, and Hup A infusion did change the hypersensitivity in both the sham and nerve-injury groups. Therefore, our data suggested that AChE in both the peripheral and central nervous systems is involved in the regulation of evoked pain.

### 4.4. Evoked Pain and Spontaneous Pain May Be Mediated by Different Mechanisms

The results of clinical studies and behavioral observations suggest that evoked and spontaneous pain are mediated by different mechanisms. Clinical studies have shown that evoked and spontaneous pain do not always coexist [14], and it has also been found that limited damage to fibers in patients changes the sensations induced by touch and warmth but may not induce spontaneous pain [29]. Here, we found that both Hup A and ambenonium chloride only alleviated mechanical allodynia but did not induce a preference for the drug-paired chamber, suggesting that spontaneous pain did not change, and these results resemble the effects of adenosine on CPP [17]. Similar results have been reported in clinical studies using ketamine, which reduces both spontaneous pain and evoked pain, whereas lidocaine only reduces evoked pain [30]. It is quite possible that evoked and spontaneous pain are regulated by different factors, and analgesic drugs that attenuate evoked pain may fail to affect spontaneous pain. Anyway, we did not find place preference induced by Hup A, and the doses that attenuated evoked pain were not enough to alleviate spontaneous pain. Therefore, other analgesic drugs should be combined with Hup A to alleviate both pain and evoked pain in clinical trials.

## Figures and Tables

**Table 1:** 

Application method	Species, strain	Pain model	Behavioral paradigm	Effects	Reference
i.t.	Rat	Formalin	Thermal escape test, formalin test	Escape latency (+), flinching (−)	[13]
i.p.	Mouse	Normal	Hot-plate test	Licking latency (+)	[11]
i.t. or i.p.	Rat (SD)	Static compression	Von Frey assay	Hindpaw-withdrawal threshold (+)	[12]

i.t., intrathecal; i.p., intraperitoneal; SD, Sprague-Dawley.
